# A 1 × 8 Optical Splitter Based on Polycarbonate Multicore Polymer Optical Fibers

**DOI:** 10.3390/s24155063

**Published:** 2024-08-05

**Authors:** Liora Lanziano, Ilay Sherf, Dror Malka

**Affiliations:** Faculty of Engineering, Holon Institute of Technology (HIT), Holon 5810201, Israel

**Keywords:** VLC, optical splitter, POF, BPM, polycarbonate

## Abstract

Visible light communication (VLC) is becoming more relevant due to the accelerated advancement of optical fibers. Polymer optical fiber (POF) technology appears to be a solution to the growing demand for improved transmission efficiency and high-speed data rates in the visible light range. However, the VLC system requires efficient splitters with low power losses to expand the optical energy capability and boost system performance. To solve this issue, we propose an effective 1 × 8 optical splitter based on multicore polycarbonate (PC) POF technology suitable for functioning in the green-light spectrum at a 530 nm wavelength. The new design is based on replacing 23 air-hole layers with PC layers over the fiber length, while each PC layer length is suitable for the light coupling of the operating wavelength, which allows us to set the right size of each PC layer between the closer PC cores. To achieve the best result, the key geometrical parameters were optimized through RSoft Photonics CAD suite software that utilized the beam propagation method (BPM) and analysis using MATLAB script codes for finding the tolerance ranges that can support device fabrication. The results show that after a light propagation of 2 mm, an equally green light at a 530 nm wavelength is divided into eight channels with very low power losses of 0.18 dB. Additionally, the splitter demonstrates a large bandwidth of 25 nm and stability with a tolerance range of ±8 nm around the operated wavelength, ensuring robust performance even under laser drift conditions. Furthermore, the splitter can function with 80% and above of the input signal power around the operated wavelength, indicating high efficiency. Therefore, the proposed device has a great potential to boost sensing detection applications, such as Raman spectroscopic and bioengineering applications, using the green light.

## 1. Introduction

Polymer optical fiber (POF) can appropriately replace traditional glass fiber [1,2]. POF offers additional benefits such as greater flexibility, lighter weight, and increased durability to vibration in unstable environments. Sensors based on optical fibers have numerous advantages compared to fiberglass due to their significant properties, including portability, remote sensing capability, and corrosive resistance [3,4,5,6]. Moreover, POF offers a variety of advantageous properties, including low cost and chemical compatibility. POF can be used in automation systems, audio and video transmission in vehicles, and in construction [7].

Polymer fibers comprise a polymer core that can transfer signals at the speed of light [8]; this core is coated with cladding that can be made of a polymer or a perfluorinated material. Another type of optical fiber that has a similar structure to POF, photonic crystal fiber (PCF), represents a versatile optical structure meticulously engineered for transmitting light. Within this structure, there are air holes situated against the background of a specific material. Light propagates through the structure in two ways: the index-driven approach and the photonic bandgap effect [9]. In the index-driven PCF, the core material possesses a higher refractive index in comparison to the cladding material. This differential refractive index gradient plays a pivotal role in guiding light through the core via the principle of total internal reflection [10,11,12,13].

The fabrication process of POF involves selecting materials for the core and cladding, typically using polymethyl methacrylate for the core and a fluorinated polymer for the cladding. A polymer preform is created by casting or extruding the core material and applying the cladding around it. The preform is heated in a drawing tower to soften the polymer and then pulled into a thin fiber while maintaining its structure. The drawing speed and temperature are controlled for desired fiber properties. The fiber is coated with a UV-curable protective layer and tested for attenuation, numerical aperture, and mechanical strength. This process results in flexible, lightweight optical fibers suitable for data communication, sensing, and illumination, allowing high-volume production at low cost [14]. To increase the efficiency of the POF, multi-core polymer optical fibers (MC-POFs) utilize the advantages of ordinary polymer single-core fibers. Thus, they consist of many cores that are arranged within a circular shape filled with cladding material [15]. Those fibers utilize the photonic bandgap alongside total internal reflection mechanisms, which enable the MC-POF to increase its transmission bitrate [16].

The main disadvantage of designing POF splitters is the light coupling mechanism, which requires a large coupling length for coupling light between two close POF cores, such as in Y-tunable POF multimode splitters and SDM fiber beam splitters [17,18]. This results in a large footprint size for the POF splitter, negatively affecting its performance in terms of energy and cost. To overcome this issue, we utilized a new light coupling mechanism based on PC MC-POF technology. This allows for a compact coupling length, resolving this issue and enabling the implementation of a more compact POF splitter.

The field of photonics has made tremendous advancements in recent years, particularly in the domain of visible light communication (VLC) based on optical components. This technology enables the transfer of large amounts of information at the speed of light, facilitating smooth and rapid communication [19]. As a result, it optimizes the existing communication infrastructure and brings about significant improvements in data transmission and connectivity [20,21]. The use of PCF structures is already ubiquitous in biomedicine [22], sensor technologies [23,24,25], lasers [26], splitters [27], and amplifiers [28] electronic components such as receivers and demultiplexers devices [29,30]. Similarly, in one example, POF splitters are used in endoscopic devices to split the green wavelength and transmit it along multiple optical fibers for illumination and imaging purposes [31]. In addition, POF splitters are used in robotic systems to distribute optical signals to different sensors and communication modules, enhancing the robot’s sensing and communication capabilities [32]. POF splitters have significant potential in various sensing applications beyond optical communication and VLC systems. They can be utilized in refractive index sensing, which is crucial for detecting changes in the refractive index of surrounding media, valuable in biochemical and environmental monitoring [33]. The sensitivity of POF splitters to different chemicals makes them suitable for chemical sensing applications, detecting the presence and concentration of various substances [34]. Additionally, POF splitters can be employed in liquid-level sensors to measure the level of liquids in containers, providing accurate and real-time data essential for industrial and biomedical fields [35].

Optical splitters are an important component of almost all optical communication systems; they assist users in making the best use of optical networks by increasing the transmission bitrate and by providing high network reliability [36,37]. Optical spitters divide the signal that is received into channels carried by the same wavelength. Specifically, we used a 530 nm wavelength that has numerous applications—for example, Raman spectroscopy uses this wavelength to identify chemical compositions and characterize materials such as toluene and chlorobenzene liquids [38]. Another use for this specific wavelength is phototherapy, which penetrates the skin to a moderate depth for therapeutic purposes. These characteristics act as significant catalysts for pioneering technological progress, especially in the field of biomedical applications [39,40,41,42].

In this work, we integrated the advantages of those optical components by presenting a new design for a PC MC-POF splitter placed on a cyclic transparent optical polymer (CYTOP) background. Strategic air holes were replaced by 23 layers of PC to manipulate and control the light coupling between two closer PC cores to achieve a uniform green light at a 530 nm wavelength for eight output channels. Thus, this unique splitter leverages POF technology, allowing us to utilize its full potential to transmit green-light signals in VLC systems.

## 2. PC MC-POF Splitter Structure and Theoretical

The splitter operates through a mechanism that uses a hexagonal lattice structure of MC-POF composed of air holes and PC layers along the fiber length in three dimensions along the central axis of the hexagon. The background material on the hexagonal surface is made of a cyclic transparent optical polymer called CYTOP. PC was chosen because of the low loss of transmitting light in the visible range.

**Figure 1:**
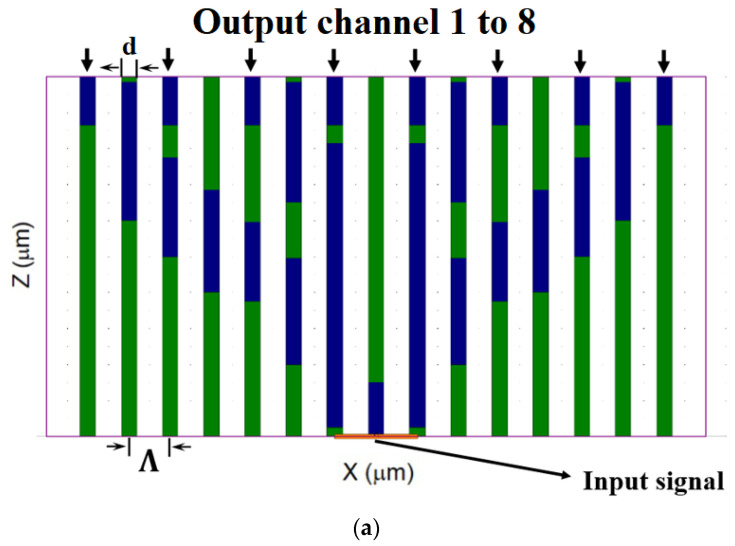

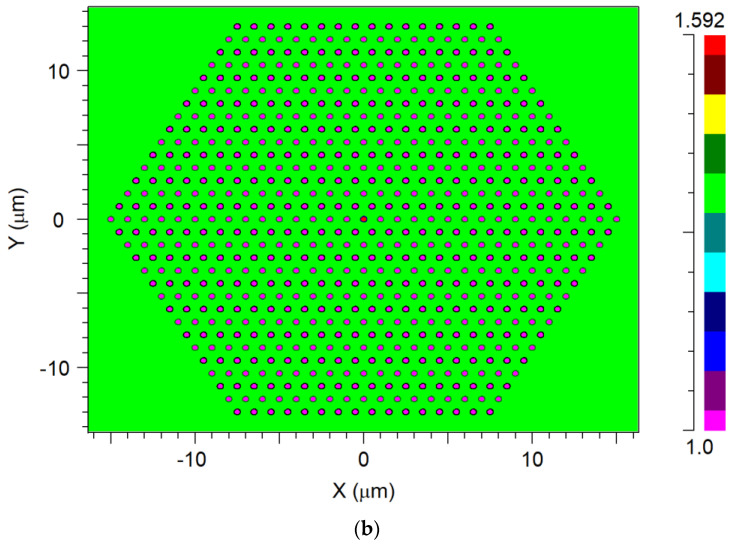
Design of the PC MC-POF 1 × 8 optical splitter operating at a wavelength of 530 nm. (**a**) Schematic sketch of the xz plane at y = 0 µm. (**b**) Refractive index profile of the splitter in the xy plane at z = 0 µm.

The refractive index values for the operated wavelength (530 nm) are n_CYTOP_ = 1.34, n_PC_ = 1.592 and n_air_ = 1. The length of the fiber is denoted as z, Λ (pitch) denotes the distance between two neighboring air holes, d denotes the diameter of the PC MC-POF structure shown in Figure 1a, and d/Λ denotes air holes diameter to pitch ratio. The optimized values of the POF geometrical parameters are z = 2 mm, d = 0.375 µm, and d/Λ = 0.375. Figure 1a shows a cross-section of the PC MC-POF splitter at the xz plane. The green areas denote air holes, the blue areas denote the PC material, and the white background denotes CYTOP. In this figure, the locations of the 23 PC layers along the length of the PC MC-POF are marked in blue. Their specific locations were set to achieve efficient energy transfer between closer PC cores, allowing the green light to be guided and split into the eight output channels effectively, thereby reducing optical power losses. The eight output channels of the input signal are shown along with the symbols of Λ and d. Figure 1b shows the cross-section of the splitter at z = 0 µm in the xy plane. The refractive index values for the operated wavelength are represented by the color bar, where the red area indicates PC, the green areas indicate CYTOP, and the pink areas indicate air holes.

The fabrication of a PC MC-POF splitter requires precise techniques to replace 23 air-hole layers with PC layers, ensuring optimal light guiding and performance characteristics. This process involves several critical steps, starting with the selection and preparation of high-quality PC material, such as Makrolon LED2245 [43], known for its superior optical clarity and mechanical strength. Initially, the PC granulates undergo thorough drying to prevent bubble formation, which could compromise optical performance. The dried granulates are then melted and cast into a cylindrical preform mold under tightly controlled temperature and pressure conditions. This casting process is crucial to achieve a homogeneous preform with minimal defects. Next, the preform is meticulously machined to create the required microstructure using high-precision drilling techniques. A hexagonal pattern of holes is carefully drilled into the preform, which is later filled with molten PC. This step is pivotal in maintaining uniformity and precision throughout the fiber. Adapting techniques akin to those used in semiconductor fabrication, the drilled holes in the preform are filled with molten PC to replace the air-filled channels traditionally used in air-hole designs. This filling process ensures uniformity and structural integrity, critical for consistent optical performance. Following the filling process, the preform undergoes a controlled drawing process to reduce its diameter and form an intermediate cane. This drawing step, conducted under precise conditions in a specialized drawing tower, maintains the integrity of the microstructure and ensures the proper formation of the PC layers. Upon achieving the desired dimensions, the intermediate cane is further drawn into a thin fiber. Throughout this final drawing stage, stringent quality control measures are implemented to monitor and optimize the PC MC-POF mechanical and optical properties. A protective polymer coating is applied during the drawing process to enhance mechanical durability and safeguard against environmental factors—a step integrated into the continuous production process. Quality control and optimization are paramount during fabrication to minimize scattering and absorption losses. Advanced techniques borrowed from CMOS fabrication, such as lithography and etching, are adapted to achieve precise microstructures in the preform. These techniques ensure the high performance and reliability of the PC MC-POF splitter.

The coupling length, which is the optical path length over the fiber necessary for successfully transferring the energy signal between two closely spaced PC layers, is defined as follows [3]:

(1) $$L_{Coupling}=\frac{\pi }{k_{0}\left(n_{symmetric}-n_{anti-symmertric}\right)},$$

*k*_0_ is the free space vector, which represents the propagation constant of the wave in free space, *n_symmetric_* is the symmetric effective refractive index, and *n_anti-symmertic_* is the anti-symmetric effective refractive index.

The beam propagation method (BPM) is used to find the coupling length more accurately by analyzing the energy transfer between two neighboring PC cores. This method solves the paraxial approximation of the Helmholtz equation, which describes how light propagates through a medium, by dividing the waveguide structure into small segments and calculating the field distribution sequentially along the propagation direction.

To compute the *L_oss_*, which represents the attenuation of the output signal for each output channel, the following equation was used [3]:

(2) $$L_{oss}=-10Log\left(\frac{p_{out}}{p_{in}}\right)\;\left(dB\right),\;$$

The output power is represented by *P_out_*, while *P_in_* denotes the input power at channel 2.

Another important property of PC MC-POF is its ability to handle the heating caused by the input laser over its lifetime, which can lead to a deviation in the operating wavelength. Consequently, the overall input wavelength shift can be described as follows [36]:

(3) $$∆\lambda \approx \lambda \times \left(-\frac{dn}{dT}\right)\times ∆T,$$

where λ is the operated wavelength of 530 nm, dT/dn represents the thermo-optic coefficient for PC with an approximate value of −1.2 × 10^−4^ per °C, and ΔT is the overall change from room temperature.

## 3. Simulation Results

The simulations and the numerical analysis used to optimize the key geometrical parameters of the PC MC-POF structure were performed by using RSoft Photonics CAD Suite software (version 2021.03), which utilized the BPM. Moreover, the BPM results were analyzed using MATLAB script codes in order to optimize and find the tolerance ranges that can support the fabrication process of the proposed splitter.

**Figure 2:**
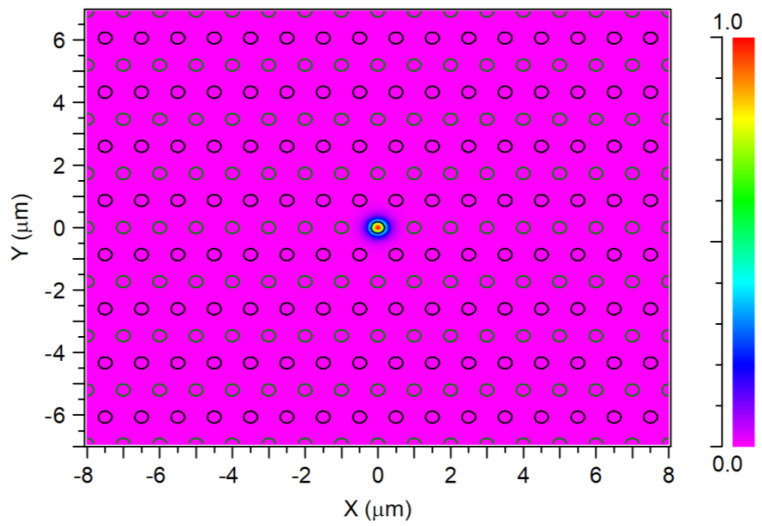
Electric-field fundamental mode solution profile at the operating wavelength of 530 nm.

Figure 2 shows the fundamental fiber mode solution for the PC MC-POF structure for the operated wavelength of 530 nm. It is important to indicate that this solution was used as the launch field at the input of our PC MC-POF structure.

**Figure 3:**
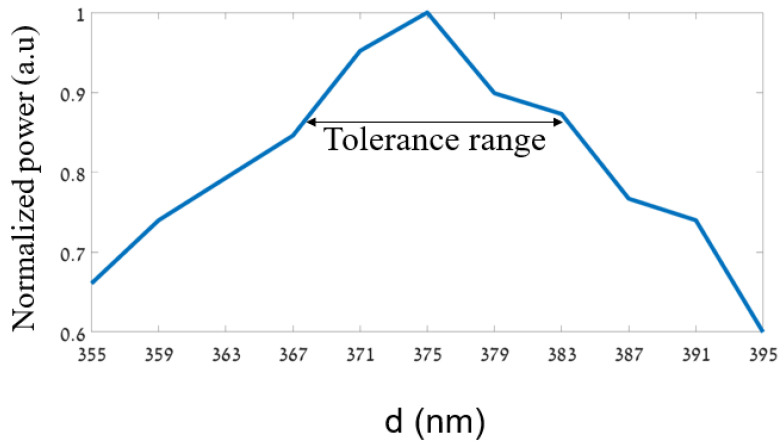
Normalized power as a function of the diameter of the MC-POF.

Figure 3 shows the optimizations of finding the optimal d value for the splitter. It can be seen from Figure 3 that the optimal value of d is 375 nm at the operated wavelength of 530 nm.

**Figure 4:**
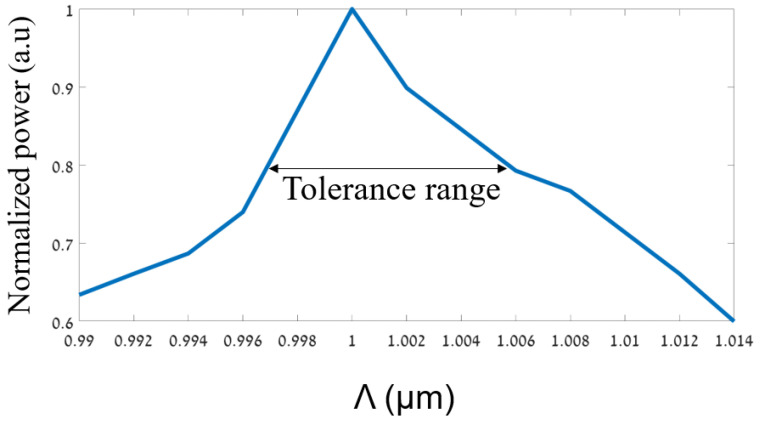
The normalized power as a function of the Λ.

Moreover, the tolerance range was analyzed to fulfill the fabrication requirement of the fiber fab, which is at least a deviation of ±10 nm from the optimal value. In our case, it can be noticed from the figure that the tolerance range is ±11 nm from the optimal value, which is suitable for transmitting 80 to 100% of the normalized power. Figure 4 shows the optimizations for finding the optimal geometrical parameter Λ. It can be seen from Figure 4 that the optimal value of Λ is 1 µm at the operated wavelength of 530 nm.

In addition, it can be noticed from the figure that the tolerance range is ±5 nm from the optimal value, which is suitable for transmitting 80 to 100% of the normalized power. Thus, this parameter has a sensitivity for the fabrication requirement of the fiber fab. To overcome this issue, a test structure of the prototype needs to be constructed to reduce the sensitivity of the pitch.

**Figure 5:**
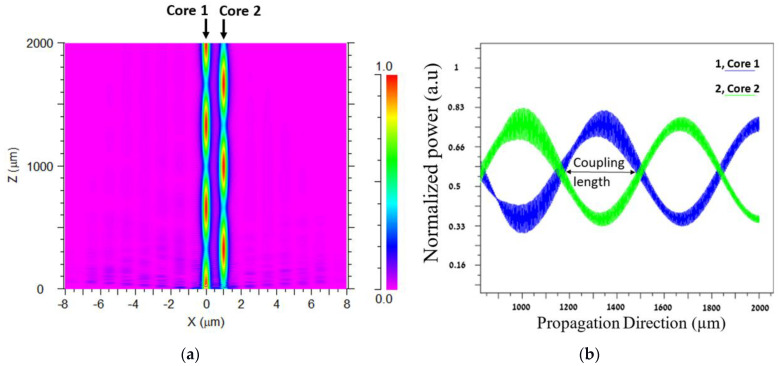
(**a**): The transfer-energy intensity profile between two adjacent cores (core 1, core 2) at the 530 nm wavelength at the xz plane; (**b**) The normalized power as a function of the direction of propagation (*z*-axis).

Figure 5a shows the transfer-energy intensity profile between two adjacent PC cores over the fiber length at the xz plane for a 530 nm wavelength. The energy intensity of the signal is represented by the color bar, where red indicates the maximum level intensity and pink indicates the minimum level intensity. In this figure, the six transfer energies between core 1 and core 2 over the fiber length from 0 to 2 mm can be seen. Figure 5b shows the mathematical sinusoidal behavior of the transfer energy and the coupling length value between two adjacent PC cores over the fiber length from 0.8 to 2 mm.

To fulfill the physical condition of Equation (1), the BPM algorithm has been used to calculate the electric field over the fiber length to reveal the coupling length size, and it can be seen from Figure 5b that the coupling length value is 341 µm (indicated by the black arrow). By finding the coupling length value at the 530 nm wavelength, the locations of the PC layers over the fiber length were located to maximize the transfer energy between closer PC cores over the PC MC-POF length and to guide the light into the eight output channels.

**Figure 6:**
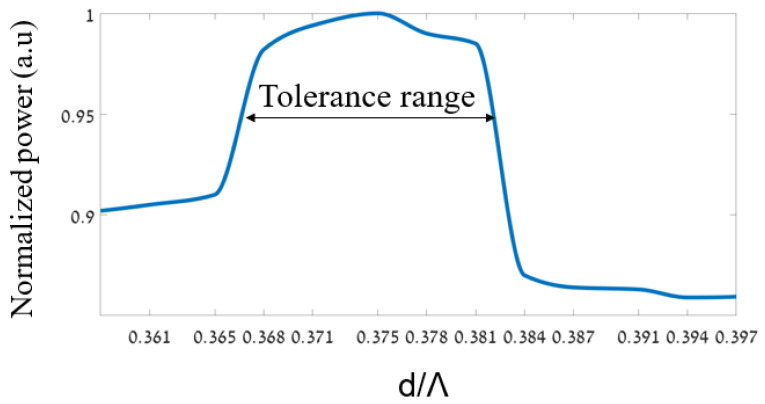
Normalized power as a function of the geometrical parameters (d/Λ) of the PC MC-POF.

Figure 6 shows the optimizations for finding the optimal geometrical parameters (d/Λ) of the PC MC-POF. It can be seen from Figure 6 that the optimal value of d/Λ is 0.375 at the operated wavelength of 530 nm.

Furthermore, the tolerance range was analyzed from the figure, and it was ±0.0144 from the optimal value, which is suitable for transmitting over 95% of the normalized power.

**Figure 7:**
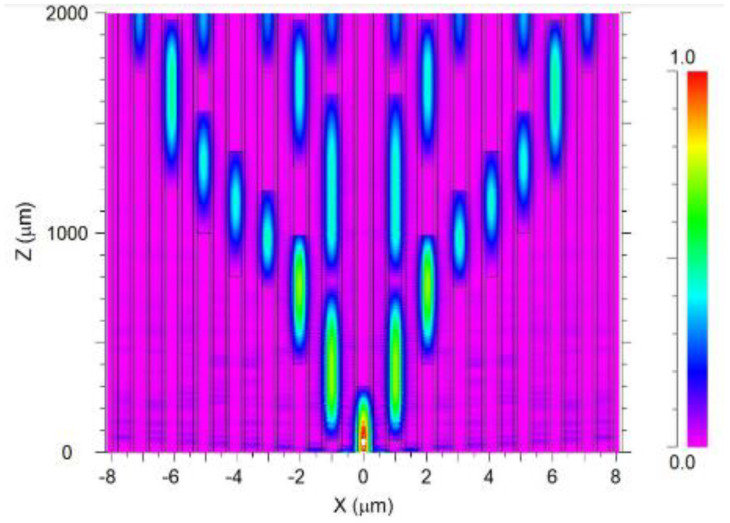
The light propagation intensity distribution over the PC MC-POF splitter.

Figure 7 shows the intensity profile and the light coupling between 23 PC layers over the fiber length at the 530 nm wavelength.

It can be seen from Figure 7 that the optical path of the input signal starts to split in two at z = 0.2 mm, and then the light is guided into four splits at z = 1.5 mm. Finally, at z = 1.8 mm, the light is split into eight and guided to the output channels at z = 2 mm.

**Figure 8:**
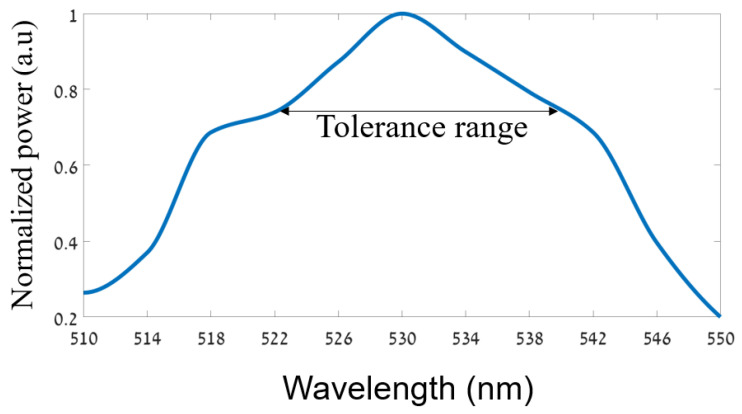
The normalized power as a function of the wavelength in the green-light spectrum.

Figure 8 shows the optical spectrum of the proposed splitter. In our case, it can be noticed from the figure that the tolerance range is ±8 nm from the operated signal, which is suitable for transmitting approximately 80 to 100% of the normalized power. Furthermore, the bandwidth is 25 nm. Thus, our proposed splitter has good stability in the green-light spectrum.

The drift effect of a laser [44], particularly in the context of a green laser, can be significantly influenced by temperature variations over its lifetime, which are typically from 25 °C to 75 °C. This effect can change the input laser wavelength, significantly reducing transmission performance. However, using Equation (3), we find that the sensitivity of the PC is 63.6 pm/°C. This means that, in the worst-case scenario, the laser can drift by 3.18 nm from the operating wavelength. Therefore, our proposed splitter demonstrates good stability against the laser drift effect due to its large tolerance range of ±8 nm from the operating signal.

To emphasize the advantages of our proposed PC MC-POF splitter, a comparison was conducted with previously published splitter devices. Table 1 compares key elements such as device footprint, ratio of splitting, power loss, operated wavelength, and the year of publication. This comparison shows that our PC MC-POF splitter has the smallest footprint and the lowest power losses, despite the large splitting ratio of eight output channels, as shown in Table 1. These advantages stem from the ability to achieve a compact coupling light length between closely cores inside the PC MC-POF, resulting in the compact size of our proposed splitter. Consequently, using our splitter can reduce energy and space costs for VLC systems. Additionally, the increased efficiency and superior performance, coupled with the unique operating wavelength of 530 nm, offer benefits for specific applications such as visible light communication and sensing, particularly in the medical field.

## 4. Conclusions

In this paper, we have proven the feasibility of designing a new green-light 1 × 8 splitter at the 530 nm wavelength based on PC MC-POF technology. This study shows how it is possible to control the light-guiding mechanism between closer PC cores by replacing 23 air-hole layers with PC layers over the fiber length, then locating them according to the coupling length size to obtain the efficient splitting of eight channels. The fabrication process for this fiber requires careful consideration. Replacing air holes with PC layers is crucial for achieving the desired light-guiding mechanism. This replacement must be carried out precisely to maintain the integrity and performance of the fiber. The results show that the splitting of eight channels happened after light coupling of 2 mm, with 0.18 dB power loss for each channel, with a large bandwidth of 25 nm. Moreover, the device has tolerance ranges for the key geometrical parameters, while the diameter has a 10 nm shift, and the pitch is more sensitive with a 5 nm shift. Thus, a test structure needs to be constructed for the prototype to fulfill the fiber fab requirement. In addition, our splitter has a large tolerance range of ±8 nm from the operated wavelength, which helps us to deal easily with the laser drift effect. The splitting into eight output channels of this proposed splitter that supports the green-light spectrum has various applications in different fields. For example, in Raman spectroscopy, the splitter can be useful by enabling the simultaneous analysis of eight different samples, drastically increasing the throughput and efficiency of Raman spectroscopy experiments. Furthermore, a splitter with eight output channels can significantly enhance the performance and versatility of VLC systems by distributing the optical power from a single light source evenly across the eight output channels, ensuring the efficient use of energy and minimizing losses. In addition to its applications in optical communication and spectroscopy, the PC MC-POF splitter operating at 530 nm green light holds significant potential as a sensor in medical biosensing. The specific wavelength of 530 nm aligns with the absorption spectra of various biomolecules and biological samples, making it suitable for sensitive detection and analysis in medical diagnostics. Medical biosensing applications can benefit from using this splitter due to its ability to efficiently split light into eight channels, each capable of carrying specific signals or interacting with different biological samples simultaneously. This capability is crucial in scenarios where multiplexed sensing is required, such as in the real-time monitoring of multiple biochemical parameters in patient samples. For instance, in biomedical laboratories, the splitter can be integrated into fluorescence-based assays where different fluorescent markers or indicators are excited at 530 nm. By distributing the excitation light across multiple channels, the splitter enables the simultaneous detection of various biomarkers or analytes present in biological samples. This enhances the throughput and efficiency of diagnostic processes, crucial for timely and accurate medical diagnoses.

## Figures and Tables

**Table 1:** Comparing the key properties between different types of fiber splitters.

Splitter Type	Device Footprint (Length × Width (mm))	Ratio of Splitting	Power Loss (dB)	Operated Wavelength (nm)	Year of Publication
Y-tunable POF multimode Splitter [17]	40 × 20	1 × 2	≤1.85	632.8	2017
Circular-to-rectangular POF converter [30]	1 × 0.785	1 × 2	≤0.7	632.8	2016
SDM fiber beam splitters [18]	12 × 19	1 × 3	≤0.5	1550	2020
Polycarbonate multicore POF	2 × 0.014	1 × 8	≤0.18	530	In this work
